# The RNA helicase DDX39 contributes to the nuclear export of spliceosomal U snRNA by loading of PHAX onto RNA

**DOI:** 10.1093/nar/gkae622

**Published:** 2024-07-16

**Authors:** Ichiro Taniguchi, Tetsuro Hirose, Mutsuhito Ohno

**Affiliations:** Institute for Life and Medical Sciences, Kyoto University, Kyoto 606-8507, Japan; Graduate School of Frontier Biosciences, Osaka University, Suita 565-0871, Japan; Graduate School of Frontier Biosciences, Osaka University, Suita 565-0871, Japan; Institute for Open and Transdisciplinary Research Initiatives, Osaka University, Suita 565-0871, Japan; Institute for Life and Medical Sciences, Kyoto University, Kyoto 606-8507, Japan

## Abstract

RNA helicases are involved in RNA metabolism in an ATP-dependent manner. Although many RNA helicases unwind the RNA structure and/or remove proteins from the RNA, some can load their interacting proteins onto RNAs. Here, we developed an *in vitro* strategy to identify the ATP-dependent factors involved in spliceosomal uridine-rich small nuclear RNA (U snRNA) export. We identified the RNA helicase UAP56/DDX39B, a component of the mRNA export complex named the transcription-export (TREX) complex, and its closely related RNA helicase URH49/DDX39A as the factors that stimulated RNA binding of PHAX, an adapter protein for U snRNA export. ALYREF, another TREX component, acted as a bridge between PHAX and UAP56/DDX39B. We also showed that UAP56/DDX39B and ALYREF participate in U snRNA export through a mechanism distinct from that of mRNA export. This study describes a novel aspect of the TREX components for U snRNP biogenesis and highlights the loading activity of RNA helicases.

## Graphical Abstract

**Graphical Abstract:**
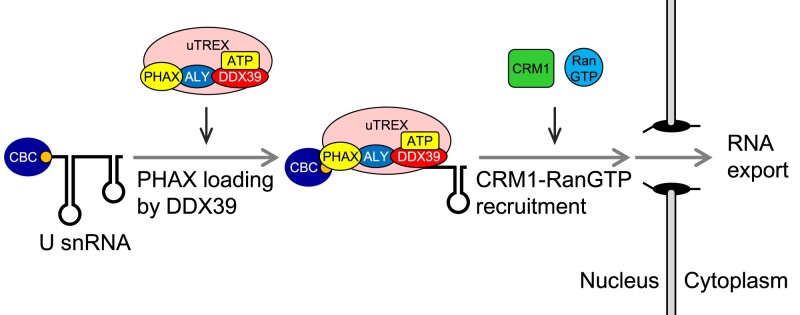

## Introduction

RNA-binding proteins dynamically interact with RNAs to regulate their function, stability, localization, and processing. The assembly/disassembly of RNA-protein complexes is mediated by RNA helicases, one of the largest classes of enzymes. RNA helicases unwind RNA duplexes and/or remove proteins from RNA in an ATP-dependent manner (1–3). These biochemical activities remodel the RNA-protein complexes. For example, during translation initiation, eIF4A/DDX2 unwinds the secondary structure of the mRNA 5′ untranslated region and allows for 43S pre-initiation complex scanning towards the start codon (4,5). DBP5/DDX19 removes nuclear RNA-binding proteins from mRNAs at the cytoplasmic filaments of the nuclear pore complex. This process releases the mRNA molecules into the cytoplasm (6–10).

In addition to RNA unwinding and protein removal, a small number of RNA helicases can reportedly load their interacting proteins onto RNA. For example, RNA helicase A/DHX9 can interact with the RNA-induced silencing complex (RISC) and load Argonaute 2 onto small interfering RNAs, thereby promoting formation of the active RISC (11,12). UAP56/DDX39B (hereafter referred to as UAP56), a component of the transcription-export (TREX) complex, loads the TREX complex onto the mRNA 7-methylguanosine (m^7^G) cap-proximal region through a direct interaction between the cap-binding complex (CBC) and the TREX component ALYREF (hereafter referred to as ALY) (13–18). SR proteins and the exon junction complex (EJC) can also bind to mRNAs. These complexes/proteins in turn recruit the mRNA export receptor TAP/NXF1-p15/NXT1 (19–26).

Similar to mRNA transcripts, nascent spliceosomal uridine-rich small nuclear RNAs (U snRNAs), with the exception of U6 and U6atac snRNAs, are synthesized by RNA polymerase II and receive the m^7^G cap structure. The adaptor protein PHAX is associated with the cap-proximal region of U snRNAs through a direct interaction with both CBC and RNA. It then recruits the nuclear export receptor CRM1-RanGTP via its nuclear export signal (NES), which promotes the export of U snRNAs to the cytoplasm (27). In the cytoplasm, the Sm complex assembly on the Sm site of U snRNAs is mediated by the survival motor neuron (SMN) complex, which contains RNA helicase GEMIN3/DDX20 (28–36). This is followed by cap trimethylation (37–39). The Sm core and the trimethylated cap structure are recognized by the import receptor complex, containing Snurportin-1 and Importin β (40,41). After being imported into the nucleus, U snRNAs are further modified and bound by specific proteins to become U small nuclear ribonucleoproteins (U snRNPs) that are responsible for pre-mRNA splicing (42,43). Among the processes involved in U snRNP biogenesis, it remains unclear if an RNA helicase is involved in the formation of the U snRNA export complex.

There are currently limited studies on RNA helicases that load their interacting proteins onto RNAs. To generalize the loading function of RNA helicases, we developed an *in vitro* strategy to identify the specific RNA helicases involved in the formation of RNA-protein complexes, focusing on the U snRNA export complex. Here, we identified UAP56 as the RNA helicase that loads PHAX onto U snRNAs. We also found that UAP56 and ALY, both of which are components of the TREX complex, are involved in U snRNA export, although mRNAs and U snRNAs are exported by distinct pathways.

## Materials and methods

### DNA constructs

The constructs for dihydrofolate reductase (DHFR) mRNA, fushitarazu (ftz) mRNA, U1ΔSm snRNA, U5ΔSm snRNA, U6Δss snRNA and tRNA^Phe^ have been previously described (44–49).

To generate the His-T7-PHAX plasmid (pET28a-PHAX), pGEX-6p-1-PHAX (50) was digested with BamHI and XhoI (TAKARA), and the full-length PHAX fragment was inserted into the same sites of pET-28a with Ligation high (TOYOBO). To generate the FLAG-ALY plasmid (pGEX-6p-1-FLAG-ALY), pGEX-6p-1-ALY (51) was digested with BamHI and XhoI, and the full-length ALY fragment was inserted into the same sites of pGEX-6p-1-FLAG-UAP56 (51) with Ligation high. To generate the FLAG-URH49 and FLAG-DBP5 plasmids (pGEX-6p-1-FLAG-URH49 and pGEX-6p-1-FLAG-DBP5, respectively), the full-length fragments were cloned and inserted into the BamHI and XhoI sites of pGEX-6p-1-FLAG-UAP56 with XE cocktail (52).

### Expression of recombinant proteins

Escherichia coli BL21 (DE3) strain bacteria were transformed with plasmids, then expression was initiated by the addition of 0.2 mM isopropyl β-d-1-thiogalactopyranoside when the culture density reached 0.6 (OD_600_). Proteins were expressed at 30°C for 3 h for recombinant RNA helicases or at 20°C overnight for recombinant PHAX and ALY proteins.

### Purification of recombinant proteins

To purify glutathione *S*-transferase (GST)-PHAX (50), cells expressing GST-PHAX were harvested and lysed in a French press in buffer-500 (20 mM Tris–HCl [pH 8.0], 0.5 M NaCl, 1 mM DTT, 10% glycerol and 0.1 mM EDTA) containing 0.1% Nonidet *P*-40 and a proteinase inhibitor. The supernatant after centrifugation was bound to Glutathione Sepharose beads (Cytiva). Bound beads were washed five times with buffer-500, and bound proteins were subsequently eluted in buffer-500 containing 10 mM reduced glutathione. The eluate was dialyzed against buffer-100 (20 mM Tris–HCl [pH 8.0], 0.1 M NaCl, 1 mM DTT, 10% glycerol and 0.1 mM EDTA) and was then applied to HiTrap Heparin Sepharose (Cytiva). The bound protein was eluted with a linear gradient of NaCl (0.1–1 M). The eluate was dialyzed against buffer-100.

To purify FLAG-UAP56 wild-type (WT) protein, FLAG-UAP56 K95E mutant (51), FLAG-URH49 and FLAG-DBP5, cells expressing GST-FLAG-UAP56 WT, GST-FLAG-UAP56 K95E, GST-FLAG-URH49 and GST-FLAG-DBP5 were harvested and lysed in a French press in buffer-500 containing 1 mM EDTA, 0.1% Nonidet *P*-40, and a proteinase inhibitor. The supernatant after centrifugation was bound to Glutathione Sepharose beads. Bound beads were washed five times with buffer-500 containing 1 mM EDTA and twice with buffer-100. Bound proteins were subsequently treated with 2 U/μl of PreScission Protease (Cytiva) at 4°C overnight. The supernatant was applied to HiTrap Q Sepharose (Cytiva). The bound protein was eluted with a linear gradient of NaCl (0.1–1 M). The eluate was dialyzed against buffer-100.

To purify GST-ALY (51), cells expressing GST-ALY were harvested and lysed in a French press in buffer-500 containing 0.1% Nonidet *P*-40 and a proteinase inhibitor. The supernatant after centrifugation was bound to Glutathione Sepharose beads. Bound beads were washed five times with buffer-500, and bound proteins were subsequently eluted in buffer-500 containing 10 mM reduced glutathione. The eluate was dialyzed against buffer-500.

To purify FLAG-ALY, cells expressing GST-FLAG-ALY were harvested and lysed in a French press in buffer-500 containing 0.1% Nonidet *P*-40 and a proteinase inhibitor. The supernatant after centrifugation was bound to Glutathione Sepharose beads. Bound beads were washed five times with buffer-500. Bound proteins were subsequently treated with PreScission Protease at 4°C overnight. The supernatant was diluted with ten volumes of buffer-100 and was applied to HiTrap Heparin Sepharose. The bound protein was eluted with a linear gradient of NaCl (0.1–1 M). The eluate was dialyzed against buffer-500.

To purify His-T7-PHAX, cells expressing His-T7-PHAX were harvested and lysed in a French press in buffer-500b (20 mM Tris–HCl [pH 8.0], 0.5 M NaCl, 1 mM 2-mercaptoethanol, 10 mM Imidazole and 10% glycerol) containing 0.1% Nonidet *P*-40 and a proteinase inhibitor. The supernatant after centrifugation was bound to Ni Sepharose beads (Cytiva). Bound beads were washed five times with buffer-500b, and bound proteins were subsequently eluted in buffer-500b containing 0.5 M imidazole. The eluate was dialyzed against buffer-100 and was then applied to HiTrap Heparin Sepharose. The bound protein was eluted with a linear gradient of NaCl (0.1−1 M). The eluate was dialyzed against buffer-100. Coomassie staining of purified recombinant proteins is shown in Supplementary Figure S1.

### *In vitro* transcription

^32^P-labeled RNAs were transcribed in a 10 μl volume containing 20 U T7 RNA Polymerase (Promega), Transcription Buffer (Promega), 1 mM DTT (Promega), 12 U RNasin Plus (Promega), NTP mixture (0.5 mM ATP, 0.5 mM CTP, 0.1 mM UTP and 0.1 mM GTP), 1 μg DNA template, 1 mM m^7^G(5′)ppp(5′)G RNA Cap Structure Analog (New England Biolabs) and 0.74 MBq of [α-^32^P] UTP (PerkinElmer). Cap Structure Analog was not added for U6Δss snRNA or tRNA^Phe^. After incubation for 60 min at 37°C, RNA was recovered from the supernatants by phenol/chloroform extraction and purified using G-50 micro columns (Cytiva). RNA was then precipitated with ethanol and dissolved in H_2_O.

### *In vitro* RNA-protein binding assay

For RNA co-immunoprecipitation (IP), ^32^P-labeled RNAs were mixed with 0.5 mM ATP, 20 mM creatine phosphate, 2 mM MgCl_2_ and 30% HeLa nuclear extracts (HNEs; Cil Biotech) and incubated at 30°C for 20 min. After centrifugation, the reaction mixture was then rotated at 4°C for 1–3 h with Protein A Sepharose beads (Cytiva) that were pre-bound to an anti-PHAX antibody (53) or anti-mouse IgG antibody (Merck). The beads were then washed five times with RSB100N buffer (10 mM Tris–HCl [pH 7.5], 0.1 M NaCl, 2.5 mM MgCl_2_ and 0.1% Nonidet *P*-40) and incubated in Homomix (50 mM Tris–HCl [pH 7.5], 5 mM EDTA, 1.5% SDS, 0.3 M NaCl, and 1.5 mg/ml proteinase K; Nacalai Tesque) at 50°C for 30 min. RNA was recovered from the supernatant by phenol/chloroform extraction and ethanol precipitation, then analyzed by denaturing polyacrylamide gel electrophoresis (PAGE) and autoradiography.

For GST pull-down, ^32^P-labeled RNAs were mixed with 0.5 mM ATP, 20 mM creatine phosphate, 2 mM MgCl_2_, 30% HNEs and either purified recombinant 50 nM GST or GST-PHAX, and the mixture was incubated at 30°C for 20 min. After centrifugation, the reaction mixture was then rotated at 4°C for 1–3 h with Glutathione Sepharose beads equilibrated with RSB100N buffer. The beads were then washed five times with RSB100N buffer and incubated in Homomix at 50°C for 30 min. RNA was recovered from the supernatant by phenol/chloroform extraction and ethanol precipitation. The samples were then analyzed by 8% denaturing PAGE and autoradiography.

To prepare nuclear lysates from *Xenopus* oocytes, 500 nuclei were dissected from *Xenopus* oocytes with sharp forceps and collected in a 1 ml volume containing 20 mM Hepes–NaOH [pH 7.9], 0.1 M KCl, 1 mM DTT, 0.2 mM EDTA and 1 mM PMSF. Nuclei were passed through a 27‐gauge needle 5 times and lysates were concentrated to 100 μl using >10 kDa Amicon Ultra (Merck). For GST pull-down, nuclear lysates from *Xenopus* oocytes were used instead of HNEs and the incubation was done at 19°C.

For RNA binding of UAP56, ^32^P-labeled RNAs were mixed with or without 2 mM nucleotide (ATP, ADP, ATP-γS), 2 mM MgCl_2_ and 2 μM FLAG-UAP56, and incubated at 30°C for 20 min. The reaction mixture was then rotated at 4°C for 1–3 h with Dynabeads Protein A (Thermo Fisher Scientific) that were pre-bound to an anti-FLAG M2 antibody (Merck). The beads were then washed five times with RSB100N and incubated in Homomix at 50°C for 30 min. RNA was recovered from the supernatant by phenol/chloroform extraction and ethanol precipitation, then analyzed by denaturing PAGE and autoradiography.

### Searching for factors that bind PHAX and ATP

The mixture was incubated in a 250 μl volume containing 200 μl HNEs, 5 μg GST-PHAX, 50 μg RNase A, 0.5 mM ATP, 20 mM creatine phosphate, and 2 mM MgCl_2_ at 30°C for 20 min. After centrifugation, the reaction mixture was added to Glutathione Sepharose beads equilibrated with RSB100N buffer and incubated on a rotating platform at 4°C for 1 h. Beads were then washed five times with buffer-150 (50 mM Tris–HCl [pH 7.5], 150 mM NaCl, 1 mM DTT, 0.2 mM EDTA, 10% glycerol). Bound proteins were subsequently treated with PreScission Protease (Cytiva) at 4°C overnight, and the supernatant was dialyzed against buffer-100. The mixture was incubated in a 20 μl volume containing the 14 μl dialysate, 0.37 MBq of [α-^32^P] ATP (PerkinElmer), and 2 mM MgCl_2_ at 30°C for 15 min. The reaction mixture was irradiated with 254 nm ultraviolet (UV) light (FUNA-UV-LINKER FS-800; Funakoshi) at 200 mJ/cm^2^ on ice. The irradiated sample was analyzed by SDS-PAGE and autoradiography. To identify the cross-linked protein, IP was performed followed by SDS-PAGE and autoradiography.

### Western blotting

Primary antibodies were used in TBS-T buffer (20 mM Tris–HCl [pH 7.5], 150 mM NaCl and 0.1% Tween 20) containing 5% skim milk. Incubations were generally performed at 4°C overnight. For secondary antibodies, horseradish peroxidase (HRP)-labeled anti-mouse, anti-rabbit, or anti-goat antibodies (Jackson ImmunoResearch) were used in TBS-T buffer containing 5% skim milk. Incubations were conducted as recommended by the manufacturers. The antibodies used in this study are described in Supplementary Table S1.

### *In vitro* protein–protein binding assays

Purified recombinant proteins were mixed with 2 mM ATP, 2 mM MgCl_2_, 1 mg/ml BSA and 0.5 mg/ml RNase A. In the presence of HNEs, purified recombinant proteins and HNEs were mixed with 0.5 mM ATP, 20 mM creatine phosphate, 2 mM MgCl_2_ and 0.5 mg/ml RNase A. The mixture was incubated at 30°C for 20 min. After centrifugation, the reaction mixture was added to Glutathione Sepharose beads equilibrated with RSB100N buffer and incubated on a rotating platform at 4°C for 1 hour. The beads were then washed five times with RSB100N buffer, then the bound material was recovered and analyzed by SDS-PAGE and western blotting.

### Identification of bridging factors between PHAX and UAP56

The mixture was incubated in a 250 μl volume containing 200 μl HNEs, 5 μg GST-PHAX, 5 μg FLAG-UAP56, 50 μg RNase A, 0.5 mM ATP, 20 mM creatine phosphate, and 2 mM MgCl_2_ at 30°C for 20 min. After centrifugation, the reaction mixture was added to an anti-FLAG M2 affinity gel (Merck) equilibrated with RSB100N buffer and incubated on a rotating platform at 4°C for 1 h. The beads were then washed five times with RSB100N buffer and bound proteins were subsequently eluted with 3xFLAG peptide. The eluate was applied to Glutathione Sepharose. The bound proteins were treated with HRV 3C protease, and the supernatant was fractionated by SDS-PAGE followed by Coomassie brilliant blue (CBB) staining. Mass spectrometry analyses of cut-out bands were entrusted to Japan Proteomics.

### *Xenopus* oocyte microinjection

*Xenopus* oocyte microinjections were performed as previously described (54). Briefly, ^32^P-labeled RNAs were injected with antibodies (200 ng IgG/oocyte) into the nucleus. After incubation at 19°C, oocytes were dissected into nuclear and cytoplasmic fractions with sharp forceps. These fractions were incubated in Homomix buffer at 50°C for 30 min. RNA was recovered from the supernatants by phenol/chloroform extraction and ethanol precipitation, then analyzed by 8% denaturing PAGE and autoradiography.

### ATPase assay

The mixture was incubated in a 20 μl volume containing 5 μM FLAG-UAP56, 10 mM Tris–HCl [pH 8.0], 0.1 M NaCl, 1 mM DTT, 5% glycerol, 5 μM ATP, 0.185 MBq of [γ-^32^P] or [α-^32^P] ATP (PerkinElmer), and 2 mM MgCl_2_ at 37°C. After incubation, the reaction was stopped in Homomix buffer at 50°C for 30 min. Products were developed by thin layer chromatography on polyethyleneimine-cellulose sheets using 0.8 M LiCl and 0.8 M acetic acid, and detected by autoradiography.

## Results

### ATP is required for efficient RNA binding of PHAX

**Figure 1.**
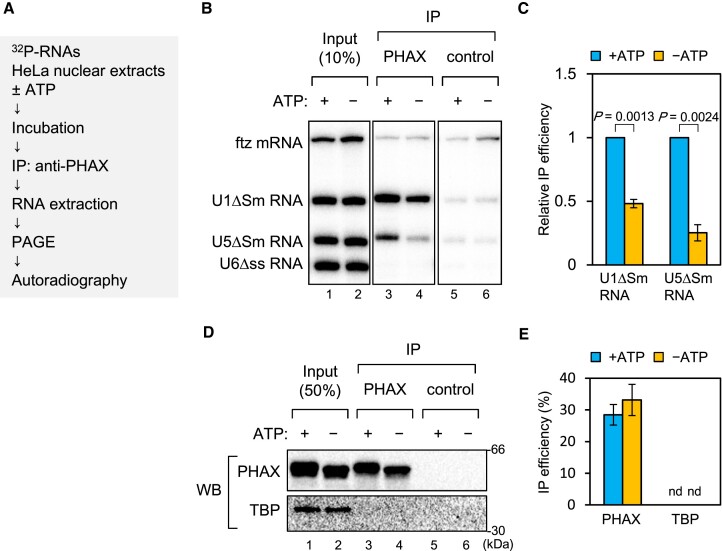
ATP is required for efficient RNA binding of PHAX in HeLa nuclear extracts (**A**) Schematic diagram of the *in vitro* PHAX-RNA binding assay. (**B**) A mixture of *in vitro*-transcribed ^32^P-labeled RNAs containing fushitarazu (ftz) mRNA, U1ΔSm, U5ΔSm and U6Δss small nuclear RNAs (snRNAs) was incubated with HeLa nuclear extracts (HNEs) in the presence or absence of ATP, then RNA co-immunoprecipitation (co-IP) assays were performed using an anti-PHAX or anti-mouse IgG antibody. U6Δss snRNA was uncapped, while the other RNAs were m^7^G-capped. Precipitated RNAs were analyzed by denaturing polyacrylamide gel electrophoresis (PAGE) and autoradiography. (**C**) The quantification of relative IP efficiency from three independent experiments is shown. IP efficiencies were determined by dividing the amount of immunoprecipitated RNA by the amount of each RNA input. Averages and standard deviations are noted. IP efficiency in the presence of ATP was set to 1. *P*-values were calculated by a two-tailed *t*-test. (**D**) Precipitated proteins were analyzed by SDS-PAGE and western blotting. ^32^P-labeled RNAs were not included in this assay. TBP: TATA-binding protein. (**E**) The quantification of IP efficiency from three independent experiments is shown. IP efficiencies were determined by dividing the amount of immunoprecipitated protein by the amount of each protein input. Averages and standard deviations are noted. nd: not detected.

RNA helicases catalyze RNA-protein complex formation in an ATP-dependent manner. To understand if RNA helicase is involved in U snRNA export, we first investigated the ATP dependency of U snRNA export complex formation. We developed an *in vitro* RNA–protein binding assay and analyzed PHAX binding to U snRNA (Figure 1A). A mixture of ^32^P-labeled *in vitro* transcribed RNAs containing intronless fushitarazu (ftz) mRNA, U1ΔSm, U5ΔSm and U6Δss snRNAs was incubated with HeLa cell nuclear extracts (HNEs) in the presence of ATP. All RNAs were m^7^G-capped, except for U6Δss snRNA. After the incubation, PHAX was precipitated with an anti-PHAX antibody, and co-precipitated RNA was analyzed by denaturing PAGE. U1ΔSm and U5ΔSm snRNAs were efficiently co-precipitated, while ftz mRNA and U6Δss snRNA were not (Figure 1B, lane 3, and C), consistent with our previous finding that PHAX preferentially binds to m^7^G-capped U snRNAs *in vivo* (53,55). When the mixture was incubated in the absence of ATP, PHAX binding to U1ΔSm and U5ΔSm snRNAs was significantly decreased (Figure 1B, lane 4, and C). Immunoprecipitation (IP) efficiency of PHAX was not affected by ATP (Figure 1D and E). These results indicated that ATP was required for the efficient RNA binding of PHAX. Moreover, they suggested that RNA helicases present in HNEs could stimulate the loading of PHAX onto the RNA.

To confirm the ATP-dependent stimulation, the same RNA mixture was incubated with purified recombinant GST-PHAX in HNEs, then a GST pull-down assay was performed in the presence or absence or ATP (Supplementary Figure S2). U1ΔSm and U5ΔSm snRNAs were efficiently pulled down with GST-PHAX in the presence of ATP. However, PHAX binding to U snRNAs was significantly decreased in the absence of ATP. Pull-down efficiency of GST-PHAX was not affected by ATP. These were consistent with the IP assay results (Figure 1).

### Identification of PHAX-interacting proteins as potential RNA helicases involved in PHAX loading

**Figure 2.**
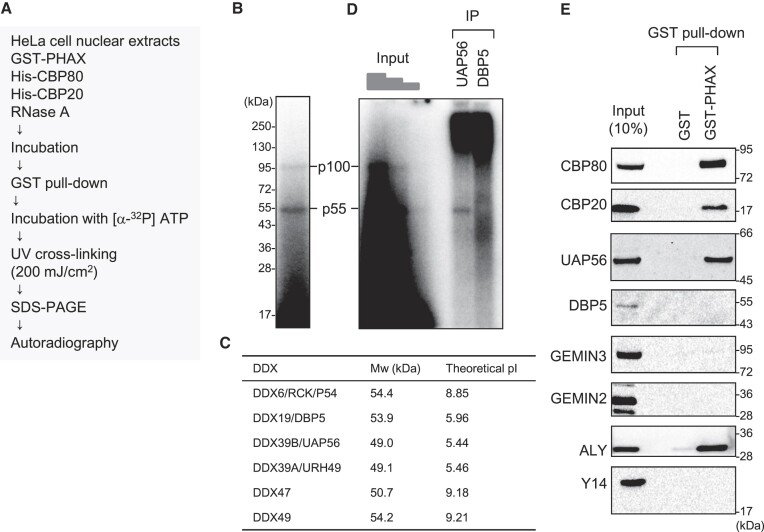
Identification of candidate helicases for ATP-dependent loading of PHAX (**A**) Schematic diagram of the process for identifying candidate helicases. HNEs were incubated with GST-PHAX, His-CBP80 and His-CBP20 in the presence of ATP and RNase A, followed by GST pull-down and HRV 3C protease treatment. PHAX-interacting proteins were irradiated by ultraviolet (UV) light and detected by SDS-PAGE and autoradiography. (**B**) The two bands corresponding to 55 kDa and 100 kDa were named p55 and p100, respectively. (**C**) The molecular weight (Mw) and theoretical isoelectric point (pI) of RNA helicases of ∼55 kDa are shown. Mw and theoretical pI were obtained from the ExPASy Compute pI/Mw tool. (**D**) The irradiated sample in (B) was immunoprecipitated using antibodies against UAP56 and DBP5, then detected by SDS-PAGE and autoradiography. (**E**) HNEs were subjected to GST pull-down assay using GST or GST-PHAX in the presence of RNase A followed by western blotting.

One of the biochemical features of RNA helicases is that they can be cross-linked to ATP by ultraviolet (UV) irradiation. For example, when purified recombinant wild-type (WT) UAP56 protein or the ATP-binding deficient mutant protein (K95E) was irradiated with UV light in the presence of ^32^P-labeled ATP, the WT protein, but not K95E, was labeled with ^32^P (Supplementary Figure S3) (51). If RNA helicase loaded PHAX onto RNA, then the helicase should interact with PHAX. Taking advantage of these two features, we developed an *in vitro* strategy to identify RNA helicases involved in PHAX loading (Figure 2A). Purified recombinant GST-PHAX was incubated with HNEs, ATP and RNase A, followed by GST pull-down. The pulled down proteins were incubated with ^32^P-labeled ATP and irradiated with UV light. Two bands corresponding to 55 kDa (p55) and 100 kDa (p100) proteins were successfully detected (Figure 2B). We focused on the stronger band, p55. From the observed expression in HeLa cells, nuclear localization, molecular weight on SDS-PAGE, and binding to anion exchange resin at pH 7.5 (Figure 2C, and Supplementary Figure S4), we presumed that p55 was UAP56 or DBP5. Therefore, we performed an IP assay to identify p55 using the cross-linked fraction. As a result, p55 was specifically precipitated using an antibody against UAP56, but not with one against DBP5 (Figure 2D), indicating that p55 was UAP56. GST pull-down and western blot analysis clearly showed the interaction between PHAX and UAP56 in HNEs (Figure 2E). We also observed an interaction between PHAX and ALY. The candidate RNA helicase for p100 was GEMIN3 since its molecular weight is 92.2 kDa and the theoretical isoelectric point is 6.49. However, the interaction of PHAX with GEMIN3 was not observed. Similarly, the interaction of PHAX with GEMIN2 or Y14 was not observed.

### UAP56 and URH49 stimulate RNA binding of PHAX

**Figure 3.**
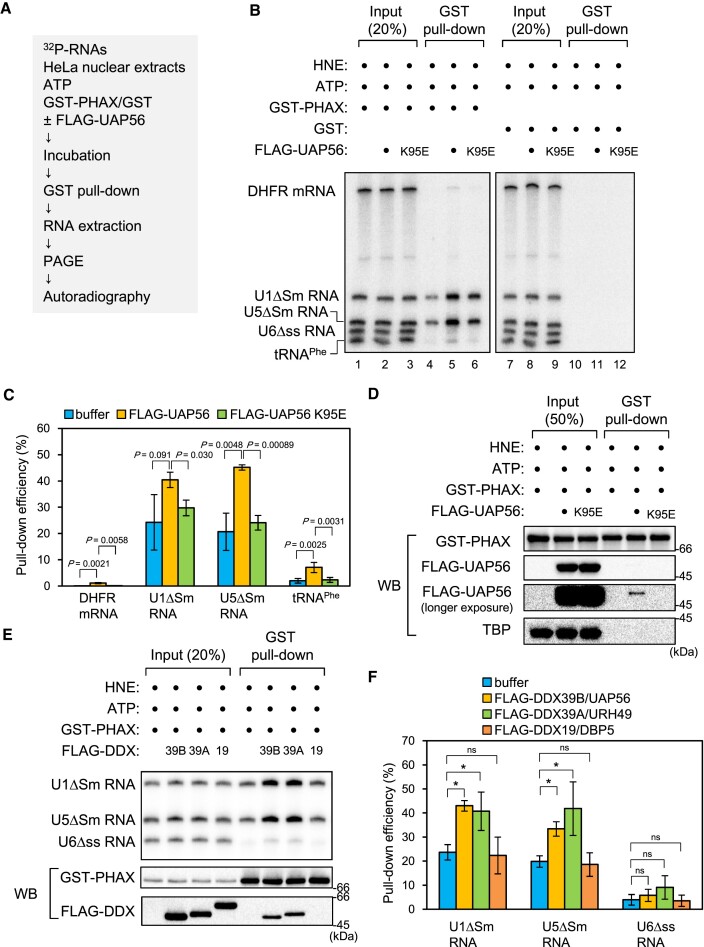
ATP-bound UAP56 stimulates RNA binding of PHAX (**A**) Schematic diagram of the *in vitro* PHAX-RNA binding assay. (**B**) A mixture of *in vitro*-transcribed ^32^P-labeled RNAs containing dihydrofolate reductase (DHFR) mRNA, U1ΔSm small nuclear RNA (snRNA), U5ΔSm snRNA, U6Δss snRNA, and tRNA^Phe^ was incubated with HeLa nuclear extracts (HNEs), ATP, either GST or GST-PHAX, and either buffer, FLAG-UAP56 WT, or K95E mutant. Then, GST pull-down assays were performed. U6Δss snRNA and tRNA^Phe^ were uncapped, while the other RNAs were m^7^G-capped. Pulled down RNAs were analyzed by denaturing PAGE and autoradiography. (**C**) The quantification of pull-down efficiency of DHFR mRNA, U1ΔSm snRNA, U5ΔSm snRNA and tRNA^Phe^ from four independent experiments performed as in (B). Pull-down efficiencies were determined by dividing the amount of pulled down RNAs by the amount of each RNA input. Averages and standard deviations are noted. *P*-values were calculated by a two-tailed *t*-test. (**D**) Pulled down proteins were analyzed by SDS-PAGE and western blotting. ^32^P-labeled RNAs were not included in this assay. TBP: TATA-binding protein. (**E**) A mixture of *in vitro*-transcribed ^32^P-labeled RNAs containing U1ΔSm, U5ΔSm, and U6Δss snRNAs was incubated with HNEs, ATP, GST-PHAX and either buffer, FLAG-UAP56 (39B), FLAG-URH49 (39A), or FLAG-DBP5 (19). Then, GST pull-down assays were performed. Pulled down RNAs were analyzed by denaturing PAGE and autoradiography. Pulled down proteins were analyzed by SDS-PAGE and western blotting. ^32^P-labeled RNAs were not included in this protein–protein binding assay. (**F**) Quantification of pull-down efficiency of U1ΔSm, U5ΔSm and U6Δss snRNAs from three independent experiments performed as in (E). Pull-down efficiencies were determined by dividing the amount of pulled down RNAs by the amount of each RNA input. Averages and standard deviations are noted. *P*-values were calculated by a two-tailed *t*-test. *P< 0.05, ns: not significant.

To investigate if UAP56 could load PHAX onto U snRNA, we performed the *in vitro* RNA-protein binding assay (Figure 3A). A mixture of ^32^P-labeled *in vitro* transcribed RNAs containing intronless dihydrofolate reductase (DHFR) mRNA, U1ΔSm, U5ΔSm, U6Δss snRNAs, and tRNA^Phe^ was incubated with purified recombinant GST-PHAX and either buffer or FLAG-UAP56 in the presence of HNEs and ATP, and a GST pull-down assay was performed. As shown in Supplementary Figure S2, GST-PHAX efficiently bound to U1ΔSm and U5ΔSm snRNAs (Figure 3B, lane 4). PHAX-binding to U1ΔSm and U5ΔSm snRNAs was stimulated by the addition of purified FLAG-UAP56 WT compared with the K95E mutant (Figure 3B and C). Pull-down efficiency of GST-PHAX was not affected by FLAG-UAP56 WT or the K95E mutant (Figure 3D). These results indicate that UAP56 loaded PHAX onto U snRNAs in an ATP-dependent manner. PHAX interacted with FLAG-UAP56 WT but not the K95E mutant (Figure 3D), suggesting that PHAX preferentially interacted with the ATP-bound form of UAP56.

UAP56/DDX39B and URH49/DDX39A are closely related RNA helicases (56) (Supplementary Figure S3B). The *in vitro* RNA-protein binding assay shows that URH49/DDX39A, but not DBP5/DDX19, also stimulated RNA binding of PHAX (Figure 3E and F). UAP56 and URH49, but not DBP5, interacted with PHAX (Figure 3E). These results suggest that the loading of PHAX onto RNA is the unique activity of the two DDX39 helicases, UAP56 and URH49. Similar results were obtained when the GST pull-down assay was performed using nuclear lysates from *Xenopus* oocytes instead of HNEs (Supplementary Figure S5A), indicating that in *Xenopus* oocytes as well as in HeLa cells, UAP56 loaded PHAX onto U snRNAs.

To test whether UAP56 could directly load PHAX onto RNA, we performed an *in vitro* RNA-protein binding assay using purified recombinant proteins in the absence of HNEs. A mixture of ^32^P-labeled *in vitro* transcribed RNAs containing U1ΔSm, U5ΔSm, and U6Δss snRNAs was incubated with purified recombinant CBC, PHAX, and UAP56 in the presence or absence of ATP or a slowly hydrolyzable ATP analog (ATP-γS), and an RNA co-IP assay was performed (Supplementary Figure S5B). UAP56 did not stimulate RNA binding of PHAX under these experimental conditions. This suggests that some factors in HNEs could be involved in the loading of PHAX onto RNA in cooperation with UAP56.

### ATP stimulates the interaction between PHAX and UAP56 in HNEs

**Figure 4.**
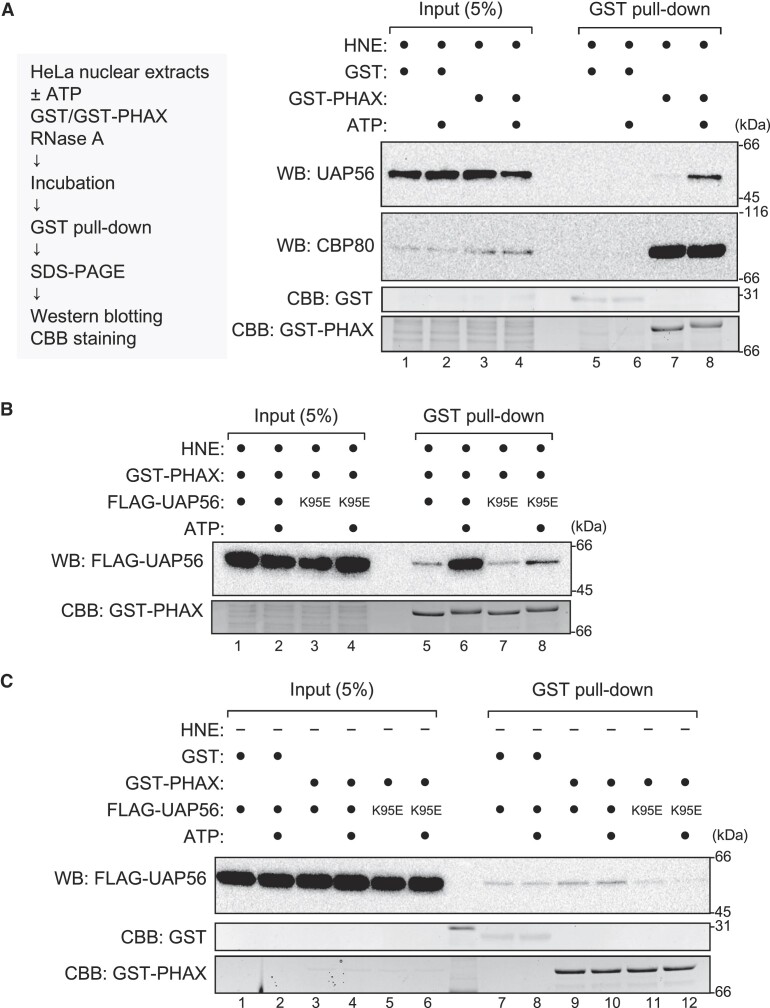
UAP56 interacts with PHAX in an ATP-dependent manner (**A**) HeLa nuclear extracts (HNEs) and RNase A were incubated with GST or GST-PHAX in the presence or absence of ATP. Pulled down proteins were detected by western blotting using anti-UAP56 and anti-CBP80 antibodies. The gel after blotting was stained with Coomassie brilliant blue (CBB). (**B**) HNEs, GST-PHAX, and RNase A were incubated with FLAG-UAP56 WT or K95E mutant in the presence or absence of ATP. Pulled down proteins were detected by western blotting using an anti-FLAG antibody. The gel after blotting was stained with CBB. (**C**) HNEs and RNase A were incubated with either GST or GST-PHAX and either FLAG-UAP56 WT or K95E mutant in the presence or absence of ATP. Pulled down proteins were detected by western blotting using an anti-FLAG antibody. The gel after blotting was stained with CBB. Uncropped CBB staining is shown in Supplementary Figure S6.

To identify the additional factors involved, we analyzed the interaction between PHAX and UAP56 in the presence of HNEs. The interaction was stimulated by the addition of ATP (Figures 4A, lanes 7 and 8, 4B, lanes 5 and 6, and Supplementary Figures S6A and B). Consistently, when the K95E mutant was used, the ATP-dependent stimulation was notably weak (Figure 4B, lanes 7 and 8, and Supplementary Figure S6B). These results suggest that PHAX preferentially interacted with the ATP-bound form of UAP56 in the presence of HNEs. To investigate whether the interaction between PHAX and UAP56 was direct, a GST pull-down assay was performed in the absence of HNEs. The interaction between PHAX and UAP56, if any, was notably weak, and its ATP dependency was not observed (Figure 4C, and Supplementary Figure S6C), indicating that the observed PHAX-UAP56 interaction in HNEs was indirect. The interaction was seen following the addition of HNEs (Supplementary Figure S6D), suggesting that some factors in HNEs mediated the interaction between PHAX and UAP56.

### Identification of bridging factors between PHAX and UAP56

**Figure 5.**
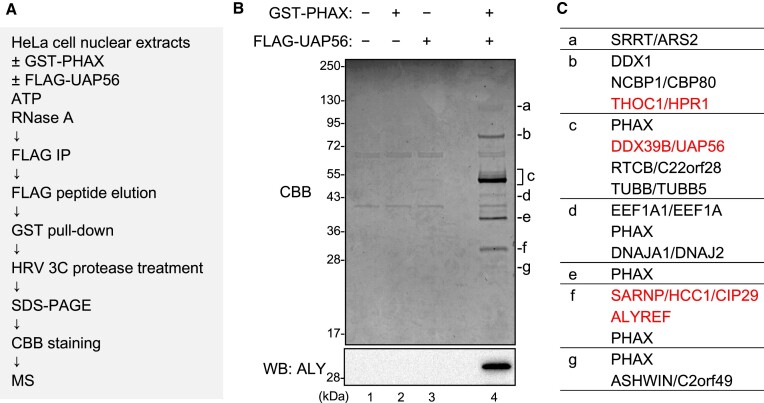
Identification of bridging factors between PHAX and UAP56 (**A**) Schematic diagram of the process for identifying bridging factors. (**B**) HeLa nuclear extracts (HNEs) and RNase A were incubated with either buffer or GST-PHAX and either buffer or FLAG-UAP56, in the presence of ATP. Immunoprecipitation (IP) experiments were then performed. The bound proteins were eluted with a FLAG peptide. The eluate was subjected to GST pull-down. The bound proteins were treated with HRV 3C protease. The supernatant was analyzed by sodium dodecyl-sulfate polyacrylamide gel electrophoresis (SDS-PAGE) and mass spectrometry or western blotting using an anti-ALY antibody. (**C**) Identified proteins specific to lane 4 of (B) are listed. Components of the TREX complex are shown in red text.

To search for the bridging factors between PHAX and UAP56, we sequentially isolated proteins that interacted with both PHAX and UAP56 from HNEs (Figure 5A). GST-PHAX and FLAG-UAP56 were incubated with HNEs, ATP, and RNase A. The mixture was applied to anti-FLAG M2 agarose to isolate FLAG-UAP56-interacting proteins. The eluate with FLAG peptide was applied to Glutathione Sepharose to isolate GST-PHAX-interacting proteins. The bound proteins were released from the beads by digesting the GST tag with HRV 3C protease, and the supernatant was fractionated by SDS-PAGE followed by CBB staining. Several specific protein bands were detected only when both GST-PHAX and FLAG-UAP56 were added to the isolation system (Figure 5B), and they were analyzed by mass spectrometry (Figure 5C, and Supplementary Table S2). Many of them were components of the TREX complex: HPR1, CIP29 and ALY (Figure 5C). Among them, ALY was a good candidate for the bridging factor because an ATP-dependent interaction between UAP56 and ALY was previously reported (57). The interaction between PHAX and ALY in HNEs was observed (Figures 2E and 5B, lower panel).

### ALY acts as a bridge between PHAX and UAP56

**Figure 6.**
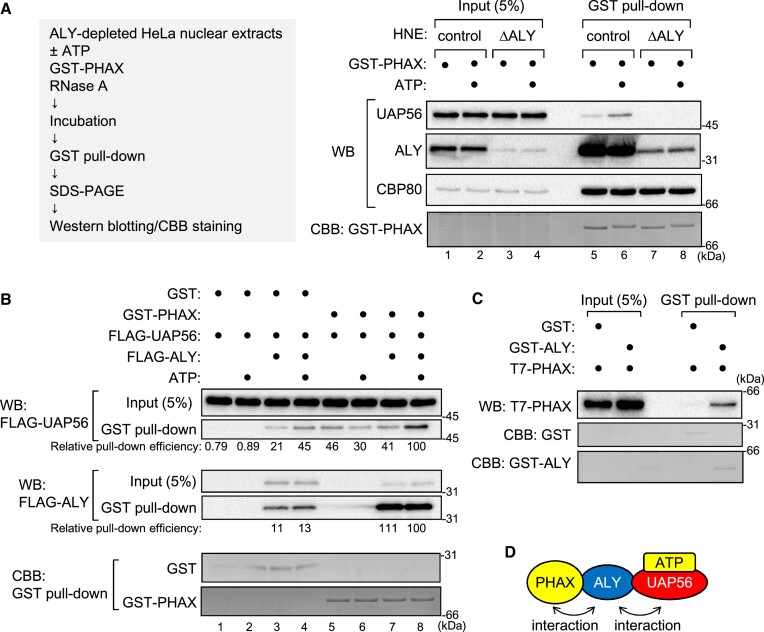
ALY bridges between PHAX and ATP-bound UAP56 (**A**) HNEs and RNase A were subjected to protein A Sepharose that was pre-bound to rabbit antibody against mouse IgG (control) or ALY (ΔALY). The depleted HNEs were incubated with GST-PHAX in the presence or absence of ATP, and GST pull-down was performed. The bound proteins were analyzed by SDS-PAGE and western blotting using antibodies against UAP56, ALY or CBP80. The gel after blotting was stained with CBB. (**B**) Purified recombinant GST or GST-PHAX was incubated with FLAG-UAP56 and RNase A in the presence or absence of FLAG-ALY and ATP, and GST pull-down was performed. The pulled down proteins were analyzed by SDS-PAGE and western blotting using an anti-FLAG antibody. The relative pull-down efficiency of FLAG-UAP56 and FLAG-ALY is shown. The gel after blotting was stained with CBB. (**C**) Purified recombinant GST or GST-ALY was incubated with T7-PHAX and RNase A, and GST pull-down was performed. Pulled down proteins were analyzed by SDS-PAGE and western blotting using an anti-T7 antibody. The gel after blotting was stained with CBB. (**D**) Schematic representation of the interaction between PHAX and UAP56 via ALY. Uncropped CBB staining is shown in Supplementary Figure S7.

When the GST pull-down assay was performed using ALY-depleted HNEs, the interaction between PHAX and UAP56 was abolished (Figure 6A, and Supplementary Figure S7A), indicating that ALY was required for the PHAX-UAP56 interaction. When the GST pull-down assay was performed in the absence of HNEs, the efficient interaction between PHAX and UAP56 was observed only when GST-PHAX, FLAG-UAP56, FLAG-ALY and ATP were all included (Figure 6B, lane 8, and Supplementary Figure S7B), indicating that ALY was sufficient for the interaction between PHAX and the ATP-bound form of UAP56. The interaction between PHAX and ALY was also observed (Figure 6B, lanes 7 and 8, and Supplementary Figure S7B). UAP56 and ALY were slightly pulled down by GST in the presence of ALY (Figure 6B, lanes 3 and 4), probably because ALY was a sticky protein and bound to GST or beads to some extent. The direct interaction between PHAX and UAP56, if any, was weak and not stimulated by ATP (Figure 6B, lanes 5 and 6). To investigate the direct interaction between PHAX and ALY, the GST pull-down assay was performed using purified GST-ALY and T7-PHAX. As expected, the interaction between PHAX and ALY was direct (Figure 6C, and Supplementary Figure S7C). These results showed that ALY could act as a bridge between PHAX and the ATP-bound form of UAP56 (Figure 6D).

### UAP56 and ALY are involved in U snRNA export

**Figure 7.**
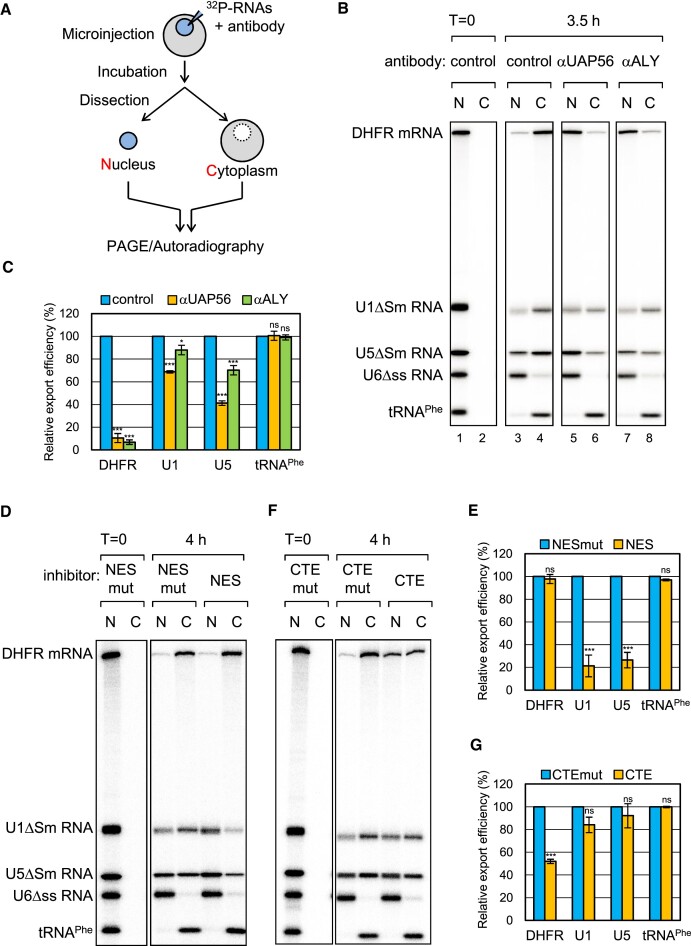
UAP56 and ALY are involved in U small nuclear RNA (snRNA) export (**A**) Schematic diagram of *Xenopus* oocyte microinjection. (**B**) The same RNA mixture as in Figure 3 was injected with a rabbit antibody against mouse IgG (control), UAP56, or ALY into the nucleus. U6Δss snRNA and tRNA^Phe^ were uncapped, while the other RNAs were m^7^G-capped. U6Δss does not leave the nucleus and served as an internal control for nuclear integrity. RNA was immediately extracted from nuclear (N) and cytoplasmic (C) fractions (lanes 1 and 2) or 3.5 h (lanes 3–8) after the injection, and then analyzed by denaturing polyacrylamide gel electrophoresis (PAGE) and autoradiography. (**C**) Quantification of the export of dihydrofolate reductase mRNA (DHFR), U1ΔSm snRNA (U1), U5ΔSm snRNA (U5), and tRNA^Phe^ from three independent experiments performed in (B). The export efficiency of the control was set to 100%. Averages and standard deviations are noted. *P*-values were calculated by comparisons with the control: *P< 0.05, ***P< 0.001, ns: not significant. (**D**) The same RNA mixture was injected with BSA-NES (NES) or BSA-NES M10 mutant (NES mut). BSA-NES, but not NES M10 mutant, saturates CRM1 export (61). RNA export was analyzed as in (B). (**E**) Quantification of the export of DHFR mRNA, U1ΔSm snRNA, U5ΔSm snRNA, and tRNA^Phe^ from three independent experiments performed in (D). The export efficiency of the control was set to 100%. Averages and standard deviations are noted. *P*-values were calculated by comparisons with the NES mut: ***P< 0.001, ns: not significant. (**F**) The same RNA mixture was injected with CTE RNA (CTE) or CTE M36 mutant RNA (CTE mut). CTE, but not CTE M36 mutant, binds specifically to TAP and inhibits the TAP-dependent export (62). RNA export was analyzed as in (B). (**G**) Quantification of the export of DHFR mRNA, U1ΔSm snRNA, U5ΔSm snRNA, and tRNA^Phe^ from three independent experiments performed in (F). Averages and standard deviations are noted. *P*-values were calculated by comparisons with the CTE mut: ***P< 0.001, ns: not significant.

UAP56 interacted with PHAX and stimulated PHAX-binding to U snRNAs in HeLa nuclear extracts and *Xenopus* oocyte nuclear lysates (Figure 3 and Supplementary Figure S5), and that ALY mediated the interaction between UAP56 and PHAX (Figure 6). To investigate if UAP56 and ALY are involved in U snRNA export, we performed the well-established *Xenopus* oocyte microinjection assay, in which RNA export is separated from other steps of gene expression and its kinetics is evaluated (e.g. (27,55,58,59) (Figure 7A). In this assay, a mixture of ^32^P-labeled RNAs containing intronless DHFR mRNA, U1ΔSm, U5ΔSm, U6Δss snRNAs, and tRNA^Phe^ was injected with an anti-mouse IgG antibody control into the nuclei of *Xenopus* oocytes. After incubation, the oocyte was dissected to separate the nucleus and cytoplasm. RNAs were then extracted from each fraction, separated using PAGE, and detected by autoradiography. All RNAs were nuclear immediately after nuclear injection (Figure 7B, lanes 1 and 2). After 3.5 h of incubation, DHFR mRNA, U1ΔSm snRNA, U5ΔSm snRNA and tRNA^Phe^ were exported to the cytoplasm, whereas the non-exported U6Δss snRNA control remained in the nucleus (Figure 7B, lanes 3 and 4). We used two antibodies against UAP56 and ALY. *In vitro* experiments show that the anti-UAP56 antibody delayed ATP hydrolysis by UAP56, and that the anti-ALY antibody inhibited RNA binding of ALY (Supplementary Figure S8). As the antibodies are polyclonal, they may have additional effects. When RNAs were injected with the anti-UAP56 antibody, DHFR mRNA export was strongly inhibited (Figure 7B, lanes 5 and 6, and Figure 7C). Notably, U1ΔSm snRNA export was slightly inhibited and U5ΔSm snRNA export was efficiently inhibited (Figure 7B, lanes 5 and 6, and Figure 7C). Similar results were obtained when using the anti-ALY antibody (Figure 7B, lanes 7 and 8, and Figure 7C). These results indicate that, in addition to mRNA export, UAP56 and ALY are involved in U snRNA export.

It has been reported that bulk mRNAs and U snRNAs are exported by different pathways, the TAP-dependent and CRM1-dependent pathways, respectively (20,27,55,60,61). However, it was thought that a fraction of U snRNAs might be exported exclusively by the TAP-dependent pathway. To examine this possibility, we used two inhibitors, BSA-NES and CTE. BSA-NES is a conjugate of NES peptides coupled to BSA that binds specifically to CRM1 (61), whereas CTE is a constitutive transport RNA element from type D retroviruses that binds specifically to TAP (62). Injection of BSA-NES and CTE inhibits the CRM1- and TAP-dependent pathways by saturating CRM1 and TAP, respectively. When RNAs were injected with BSA-NES, U1ΔSm and U5ΔSm snRNA export, but not DHFR mRNA or tRNA^Phe^ export, was strongly inhibited (Figure 7D and E). This indicates that U snRNAs, but not DHFR mRNA or tRNA^Phe^, were exported by the CRM1-dependent pathway. When CTE was injected, DHFR mRNA export, but not U snRNA or tRNA^Phe^ export, was strongly inhibited, indicating that DHFR mRNA was exported by the TAP-dependent pathway, but U snRNAs were not (Figure 7D and E). These results suggested that UAP56 and ALY participated in U snRNA export through a mechanism distinct from that of mRNA export.

We investigated the importance of UAP56 ATP binding activity in U snRNA export (Supplementary Figure S9). Consistent with previous reports (13,51), injection of WT UAP56 inhibited mRNA export in a dose-dependent manner (Supplementary Figure S9B, lanes 5–10). The UAP56 K95E mutant protein inhibited DHFR mRNA export more strongly than the WT protein (Supplementary Figure S9B, lanes 11–16) (51). Notably, the K95E mutant protein also inhibited the export of U1 and U5 snRNAs (Supplementary Figure S9). These results suggest that UAP56 and its ATP binding activity are important for export of not only mRNAs, but also U snRNAs.

### ATP, but not ADP, is required for RNA binding of UAP56

**Figure 8.**
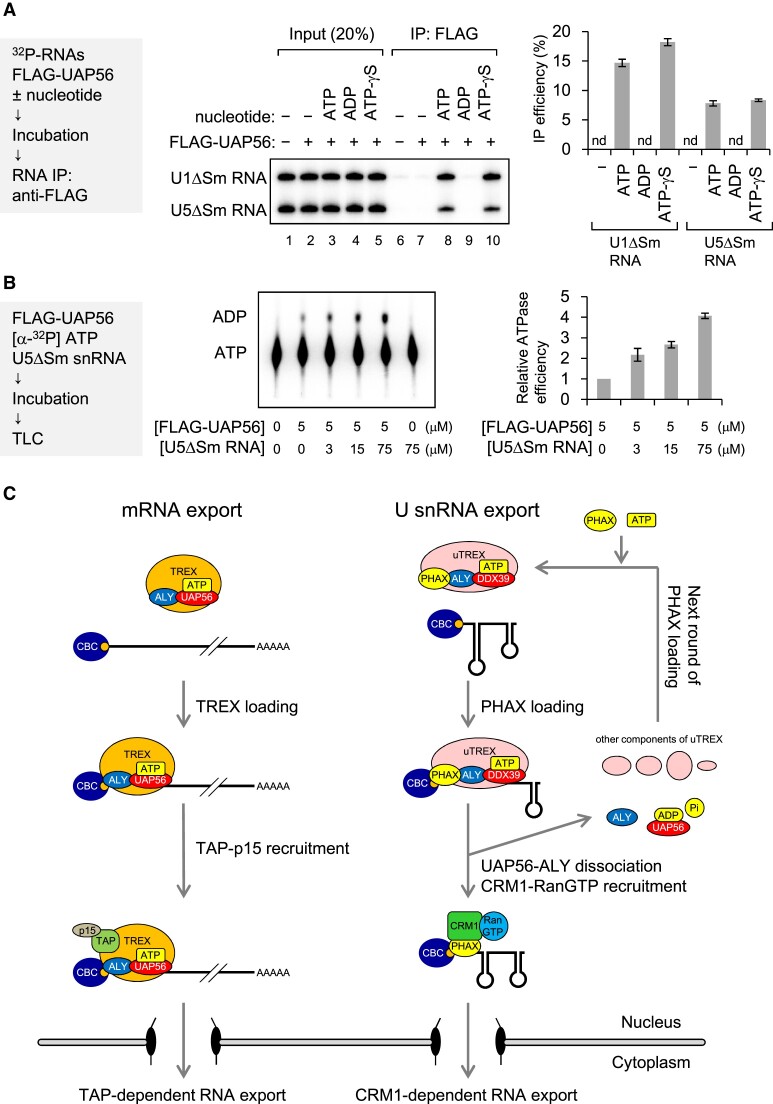
RNA binding and ATPase activities of UAP56 (**A**) *In vitro*-transcribed ^32^P-labeled U1ΔSm and U5ΔSm snRNAs were incubated with or without purified recombinant FLAG-UAP56 (1 μM) in the absence or presence of 2 mM nucleotide (ATP, ADP or ATP-γS) and 2 mM MgCl_2_, then RNA co-immunoprecipitation (co-IP) assays were performed using an anti-FLAG M2 antibody. Precipitated RNAs were analyzed by denaturing polyacrylamide gel electrophoresis (PAGE) and autoradiography. The quantification of IP efficiency from three independent experiments is shown. IP efficiencies were determined by dividing the amount of immunoprecipitated RNA by the amount of each RNA input. Averages and standard deviations are noted. (**B**) [α-^32^P] ATP was incubated with or without purified recombinant FLAG-UAP56 in the absence or presence of U5ΔSm RNA at 37°C. Products were developed by thin layer chromatography. ATP and ADP were detected by autoradiography. Quantification of ATP hydrolysis efficiency from three independent results is shown. Averages and standard deviations are noted. Relative ATPase efficiency without U5 RNA was set to 1. (**C**) A model of the mechanism by which UAP56 loads PHAX onto U small nuclear RNA (snRNA). In mRNA export, UAP56 loads the TREX complex onto mRNA and then recruits TAP-p15. UAP56 may dissociate from mRNA during TAP-p15 recruitment, leading to RNA export to the cytoplasm. Note that for the sake of simplicity, this model does not show the EJC and SR proteins as adaptors to recruit TAP-p15. In U snRNA, PHAX forms the U snRNA-type TREX complex (uTREX) with UAP56 and ALY and is loaded onto U snRNA as the uTREX complex. During the loading process, UAP56 may unwind the stable cap-proximal structure of U snRNA using energy from ATP hydrolysis. Immediately after loading and ATP hydrolysis, the uTREX complex components, except PHAX, may dissociate from RNA. The PHAX that remains then recruits CRM1-RanGTP, leading to RNA export. After dissociation, UAP56 is loaded with ATP again for the next round of loading of PHAX onto RNA.

To understand the molecular mechanism underlying PHAX loading by UAP56, we investigated RNA binding of UAP56. ^32^P-labeled *in vitro* transcribed U1ΔSm and U5ΔSm snRNAs were incubated with purified recombinant FLAG-UAP56 in the absence or presence of nucleotide (ATP, ADP and ATP-γS). After the incubation, FLAG-UAP56 was precipitated with an anti-FLAG antibody, and the co-precipitated RNA was analyzed by denaturing PAGE (Figure 8A). U1ΔSm and U5ΔSm snRNAs were efficiently co-precipitated in the presence of ATP and ATP-γS, but not in the absence of nucleotide or in the presence of ADP, suggesting that RNA binding of UAP56 required ATP, but not ATP hydrolysis. The ATPase activity of UAP56 is enhanced by several RNAs (63,64,51). U5ΔSm snRNA also enhanced the ATPase activity of UAP56 (Figure 8B). Together, these results suggest that the ATPase activity of UAP56 could be activated on U snRNA and subsequently the ADP bound-form of UAP56 could dissociate from it.

## Discussion

In this study, we developed an *in vitro* strategy to identify the RNA helicases involved in U snRNA nuclear export, which revealed that UAP56 and its highly related RNA helicase URH49 can stimulate RNA binding of PHAX. We also found that PHAX can interact with UAP56 via ALY. Both UAP56 and ALY are components of the TREX complex, and some components of the TREX complex were present in the PHAX-UAP56 interactome (Figure 5). The URH49-containing complex (AREX) is remodeled to the highly similar TREX complex upon ATP binding (65). Therefore, PHAX could be loaded as a U snRNA-type TREX-like complex (uTREX), which is composed of PHAX and several TREX components, through the ATP-dependent RNA binding activity of the RNA helicases. *Xenopus* oocyte microinjection experiments showed that UAP56 and ALY participated in U snRNA export through a mechanism distinct from that of mRNA export; U snRNA export did not involve the TAP-dependent pathway, but rather the CRM1-dependent pathway. Therefore, the uTREX components, except PHAX, likely dissociates from U snRNAs after PHAX loading, while PHAX remains on the RNA, leading to CRM1-dependent export (Figure 8C). It has been reported that UAP56 and URH49 export different subsets of mRNAs/circular RNAs through distinct complexes, the TREX and AREX complexes (66,67). Whether UAP56 or URH49 is primarily or equally involved in U snRNA export will be the focus of further studies.

Dissociation of the uTREX components, except PHAX, is likely critical for U snRNA function, because when U snRNA was artificially exported by the TAP-dependent pathway, the RNA was no longer imported into the nucleus (55). Our results suggest that the ATPase activity of UAP56 contributes to the dissociation: the ATPase activity of UAP56 could be activated on U snRNA and subsequently the ADP bound-form of UAP56 could dissociate from it (Figure 8A and B). Another probable dissociation mechanism is based on the RNA binding property of ALY. We have previously shown that UAP56 binds RNAs with broad specificity, whereas ALY can intrinsically stably bind to mRNAs but not to U snRNAs (51). Therefore, UAP56 could initially load ALY onto a wide range of RNA classes, then remaining on mRNAs but dissociating from U snRNAs. Future studies will aim to elucidate all the components of the uTREX complex and clarify the mechanism of dissociation from U snRNAs.

During the mRNA export process, UAP56 loads the TREX complex onto RNA. UAP56 also functions as an adapter between the RNA and TAP-p15 as the TREX complex. In contrast, UAP56 and ALY do not function as U snRNA export adapters. This may explain why the inhibition of mRNA export by antibodies against UAP56 and ALY and recombinant UAP56 proteins was stronger than that of U snRNA export.

U5 snRNA was more sensitive than U1 snRNA to the addition of ATP (Figure 1) and to the action of UAP56 (Figures 7 and Supplementary Figure S9). Because the U5 snRNA cap structure is located at the base of the stem-loop structure (Supplementary Figure S10), formation of the export complex at the cap structure is likely not a simple process. UAP56 may allow the loading of PHAX by unwinding the stable RNA structure near the cap, which is coupled with continuous ATP hydrolysis. For cytoplasmic U snRNA metabolism, unwinding of the cap-proximal RNA structure may also be important for the cap-binding proteins to access the cap. A good candidate RNA helicase for the loading onto the cap region would be GEMIN3, a component of the SMN complex (36).

When searching for the bridging factors between PHAX and UAP56 (Figure 5), we isolated components of the tRNA splicing-ligase complex: DDX1, RTCB, and ASHWIN (68). It would be interesting to investigate if the uTREX complex is involved in tRNA splicing. We also isolated ARS2 as the PHAX-UAP56 interacting protein. There are several reports describing that ARS2 can interact with TREX complex components, (16,57) as well as with CBC and PHAX to form the CBCAP complex (69). It is possible that ARS2 forms a larger complex containing CBC, PHAX and TREX complex components, and that this complex plays a role in the biogenesis of RNA polymerase II transcripts.

RNA helicases can remodel RNA–protein complexes through their abilities to unwind RNA structures and remove proteins from RNA. While these two activities are well studied, less is known about the direct loading of their interacting proteins onto RNA. In this study, we aimed to develop a strategy to identify RNA helicases with this loading activity, taking advantage of the property that RNA helicases can be cross-linked to ATP by UV irradiation and can interact with target proteins. Here, we successfully identified the RNA helicases UAP56 and URH49, which are involved in export factor loading onto U snRNA. The method developed in this study can be used to identify RNA helicases with loading activity in the formation of other RNA-protein complexes.

## Supplementary Material

gkae622_Supplemental_Files

## Data Availability

The data underlying this article are available in the article and in its online supplementary data. The mass spectrometry proteomics data are available at the ProteomeXchange Consortium with the dataset identifier PXD053480.
