# Supplementary material for: The RNA helicase DDX39 contributes to the nuclear export of spliceosomal U snRNA by loading of PHAX onto RNA
Source: Nucleic Acids Res. 2024 Jul 16;52(17):10668–82. doi: 10.1093/nar/gkae622 (PMC11417407; doi:10.1093/nar/gkae622)
Supplement: gkae622_Supplemental_Files [file gkae622_supplemental_files.zip › SupFiguresR2.pdf]

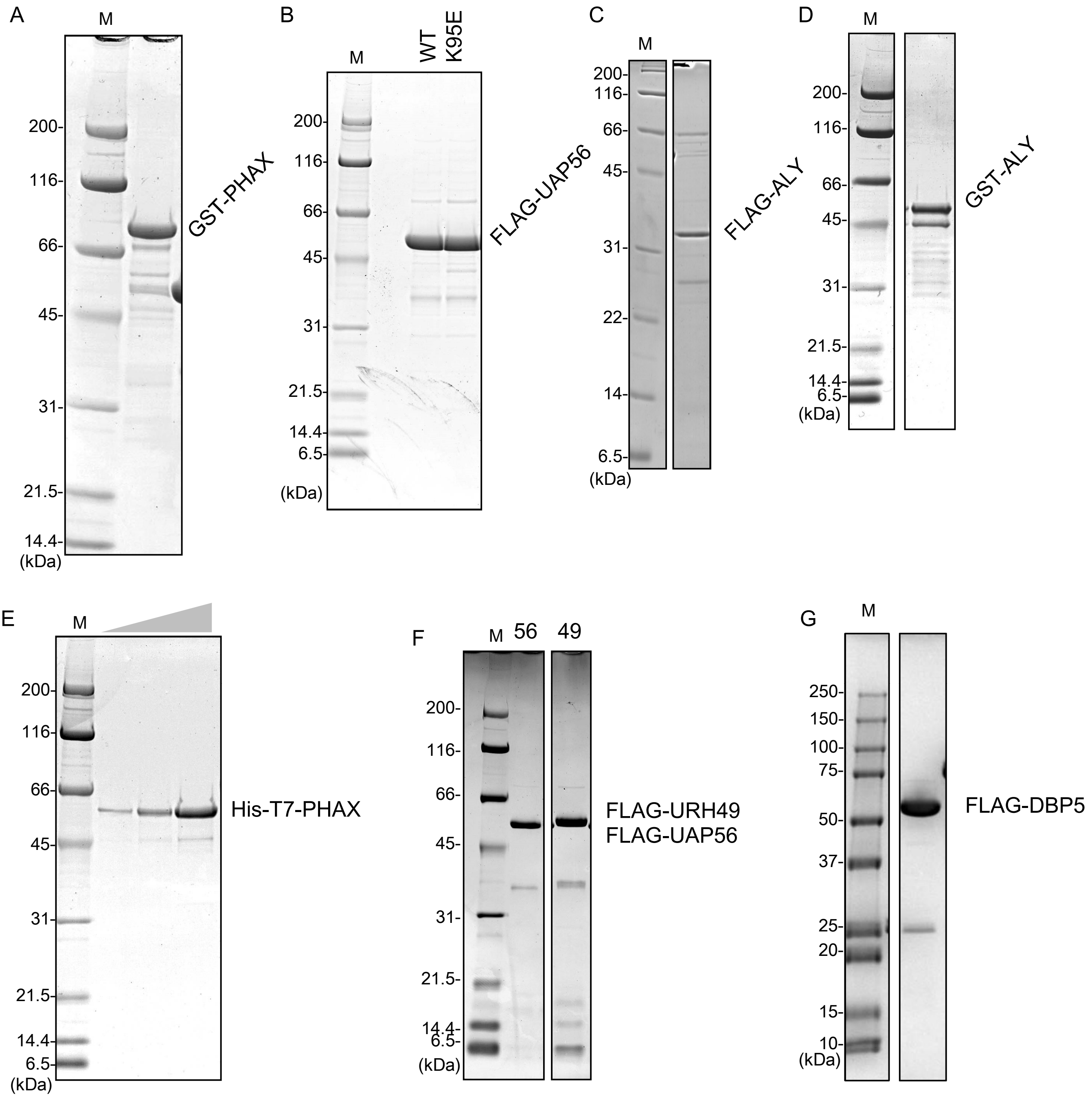

Supplementary Figure S1: Coomassie staining of purified recombinant proteins.
(A) Purified recombinant GST-PHAX.
(B) Purified recombinant FLAG-UAP56 WT and K95E mutant.
(C) Purified recombinant FLAG-ALY.
(D) Purified recombinant GST-ALY.
(E) Purified recombinant His-T7-PHAX.
(F) Purified recombinant FLAG-UAP56 (56) and FLAG-URH49 (49)
(G) Purified recombinant FLAG-DBP5.

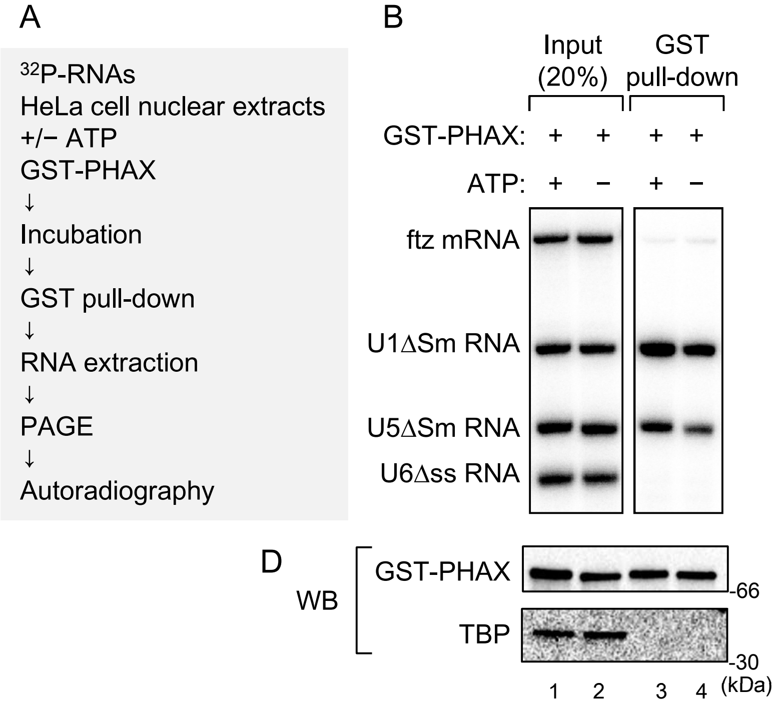

Supplementary Figure S2: The effect of ATP regeneration and hydrolysis on loading of PHAX onto U small nuclear RNA (snRNA).

(A) The same RNA mixture as in Figure 1A was incubated with HNEs and GST-PHAX in the presence or absence of ATP, and GST pull-down assays were performed.

(B) Pulled down RNAs were analyzed as in Figure 1.

(C) Quantification of relative pull-down efficiency from three independent experiments is shown. Pull-down efficiencies were determined by dividing the amount of pulled down RNAs by the amount of each RNA input. Averages and standard deviations are noted. Pull-down efficiency in the presence of ATP was set to 1. *P*-values were calculated by a two-tailed *t*-test.

(D) Pulled down GST-PHAX protein was analyzed. ^{32}P-labeled RNAs were not included in this assay. 50% of input proteins were loaded.

(E) Pull-down efficiencies were determined by dividing the amount of pulled down RNAs by the amount of each RNA input. Averages and standard deviations are noted.

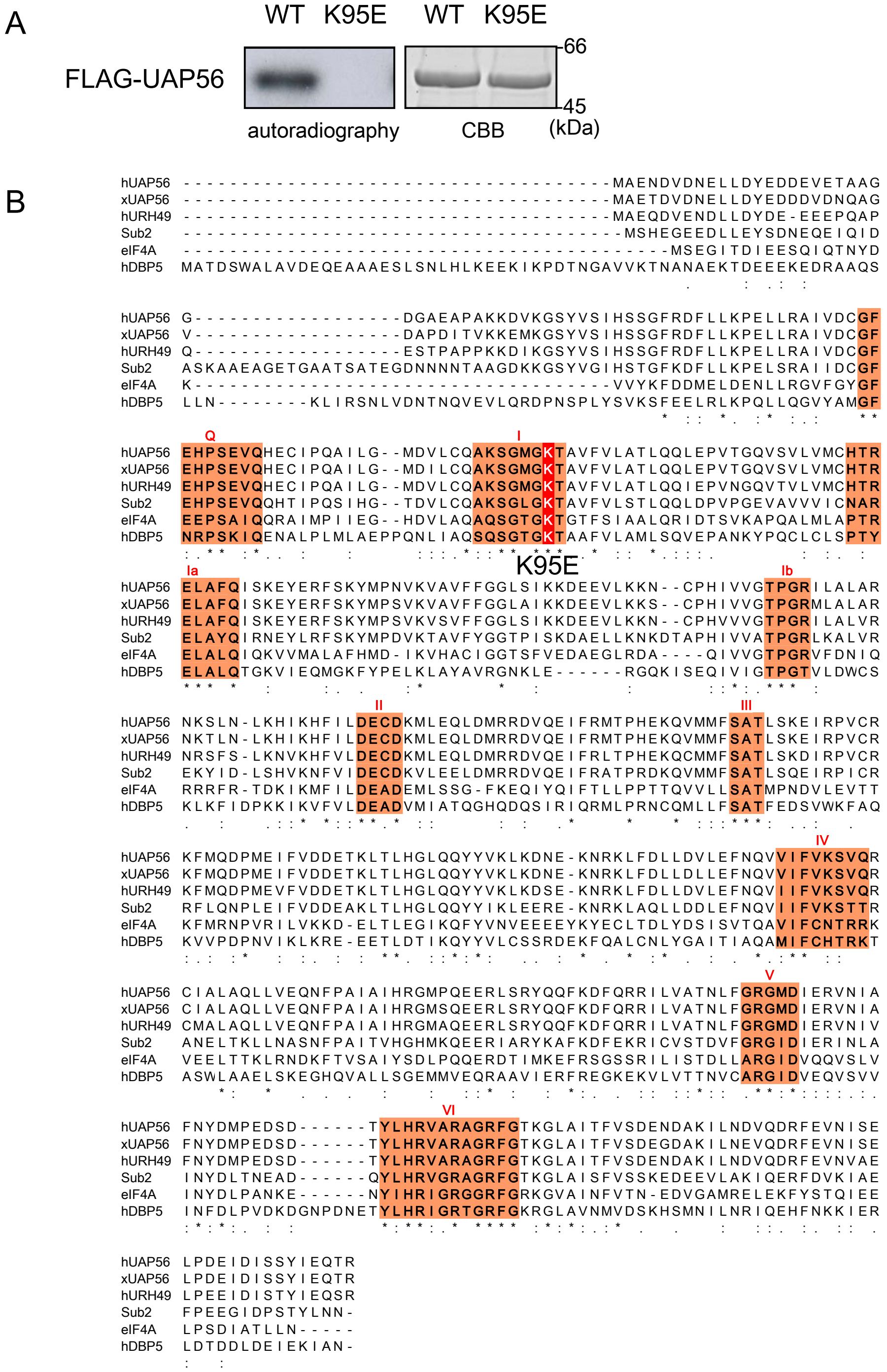

Supplementary Figure S3: ATP-binding deficient mutant of UAP56 (K95E).

(A) Purified FLAG-UAP56 WT or K95E was incubated with ^{32}P-ATP, followed by UV irradiation. Proteins were analyzed by SDS-PAGE and autoradiography/CBB staining.

(B) Multiple sequence alignment was generated using ClustalW for human UAP56 (hUAP56), *Xenopus* UAP56 (xUAP56), human URH49 (hURH49), yeast UAP56 (Sub2), yeast eIF4A, and human DBP5 (hDBP5) Conserved motifs of RNA helicases are indicated with red boxes.

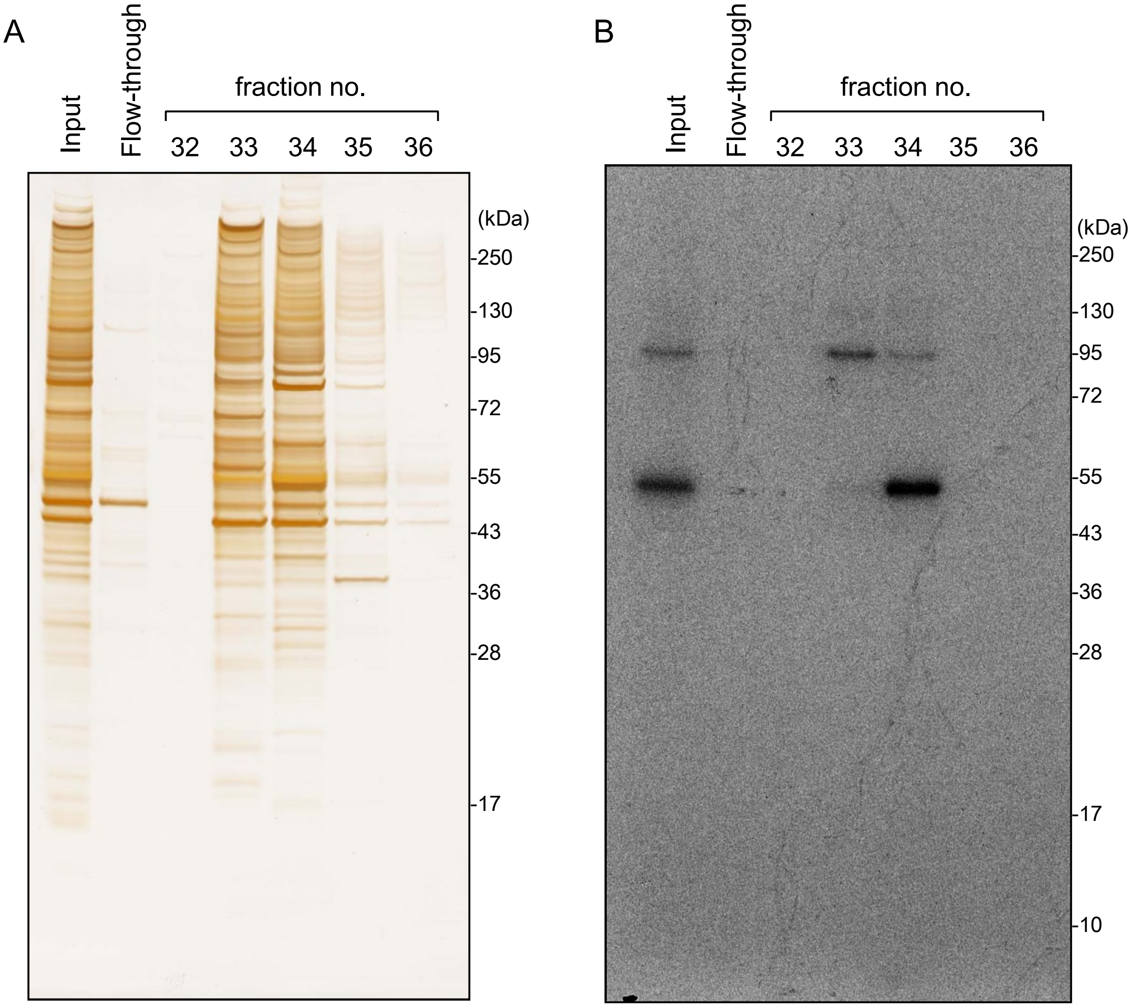

Supplementary Figure S4: Proteins that bind both PHAX and ATP.

(A) PHAX-interacting proteins were applied to Mono Q. Input, flow-through, and bound fractions were separated by sodium dodecyl-sulfate polyacrylamide gel electrophoresis (SDS-PAGE) and detected by silver staining.

(B) PHAX-interacting proteins in (A) were cross-linked with ^{32}P-ATP and separated by SDS-PAGE and detected by autoradiography.

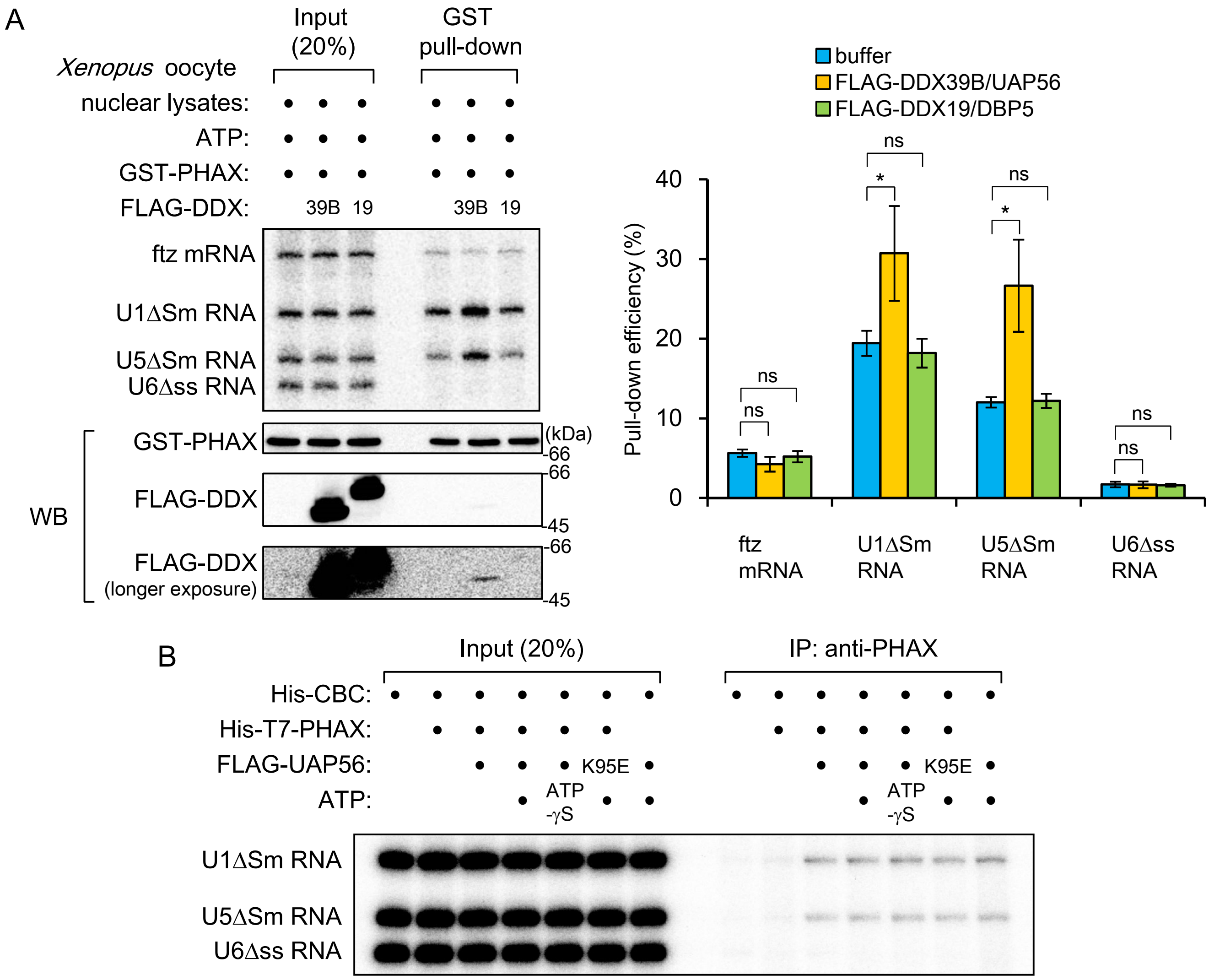

Supplementary Figure S5: Effect of RNA helicase on RNA binding of PHAX.
(A) ^{32}P-labeled ftz mRNA, U1ΔSm, U5ΔSm, and U6Δss snRNAs were incubated with purified recombinant GST-PHAX and either buffer, FLAG-UAP56 (39B), or FLAG-DBP5 (19) in the presence of nuclear lysates from *Xenopus* oocytes and ATP. Then, the GST pull-down assay was performed. Pulled down RNAs were analyzed by denaturing PAGE and autoradiography. The quantification of pull-down efficiency of each RNA from four independent experiments. Pull-down efficiencies were determined by dividing the amount of pulled down RNAs by the amount of each RNA input. Averages and standard deviations are noted. *P*-values were calculated by a two-tailed *t*-test. *$P < 0.05$, ns: not significant. Pulled down proteins were analyzed by SDS-PAGE and western blotting (WB). ^{32}P-labeled RNAs were not included in this protein-protein binding assay. 50% of input proteins were loaded. .
(B) ^{32}P-labeled U1ΔSm, U5ΔSm, and U6Δss snRNAs were incubated with His-CBP80 and His-CBP20 (His-CBC), either buffer or His-T7-PHAX, and either buffer, FLAG-UAP56 WT, or K95E mutant in the presence or absence of either ATP or ATP-γS. Then, an RNA co-immunoprecipitation assay was performed. Precipitated RNAs were analyzed by denaturing PAGE and autoradiography.

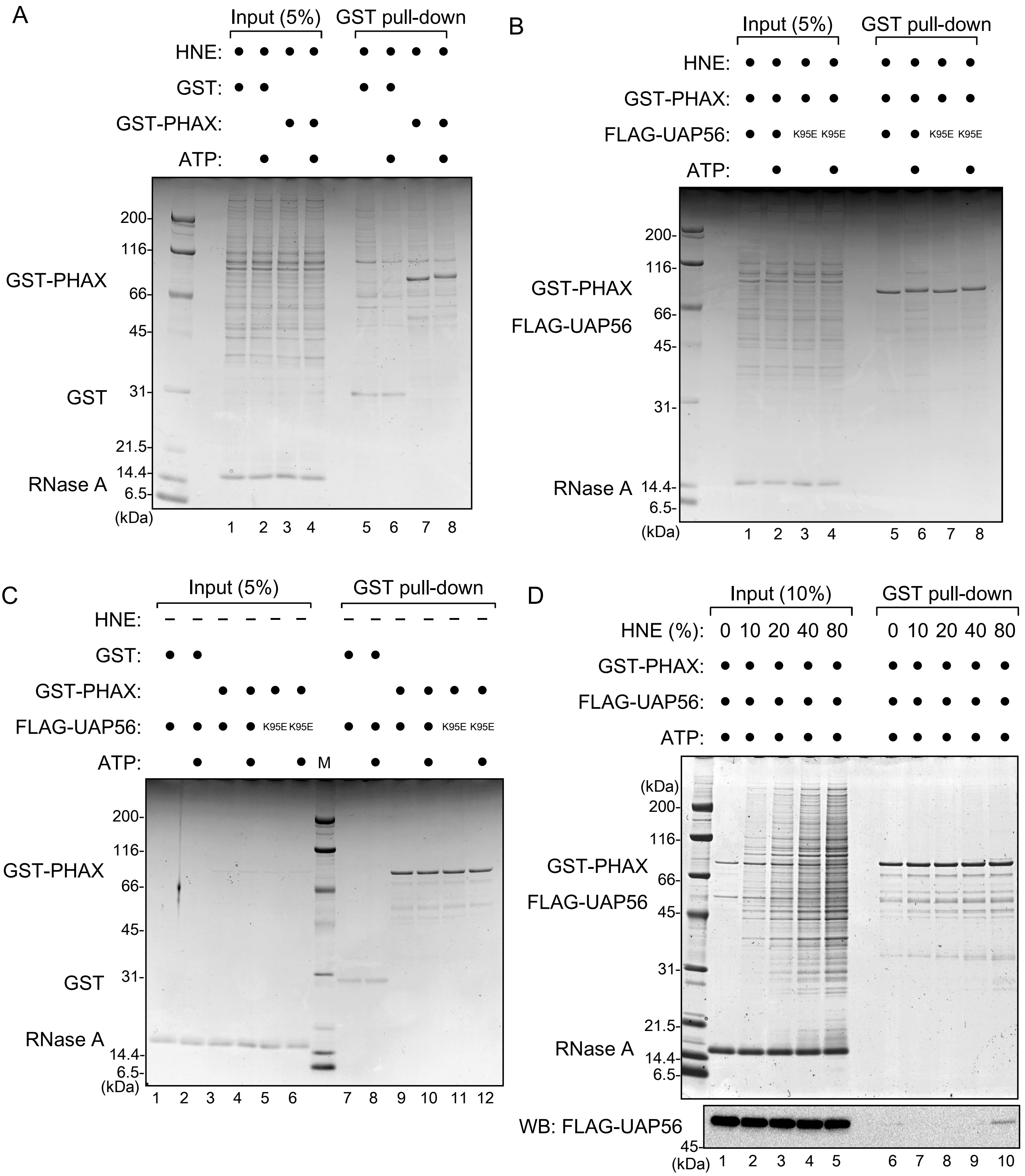

Supplementary Figure S6: Coomassie brilliant blue (CBB) staining of Figure 4.

(A—C) CBB staining of Figures 4A, 4B, and 4C.

(D) GST-PHAX, FLAG-UAP56, ATP, and RNase A were incubated with or without HNEs. Pulled down proteins were detected by western blotting using an anti-FLAG antibody. The gel after blotting was stained with CBB.

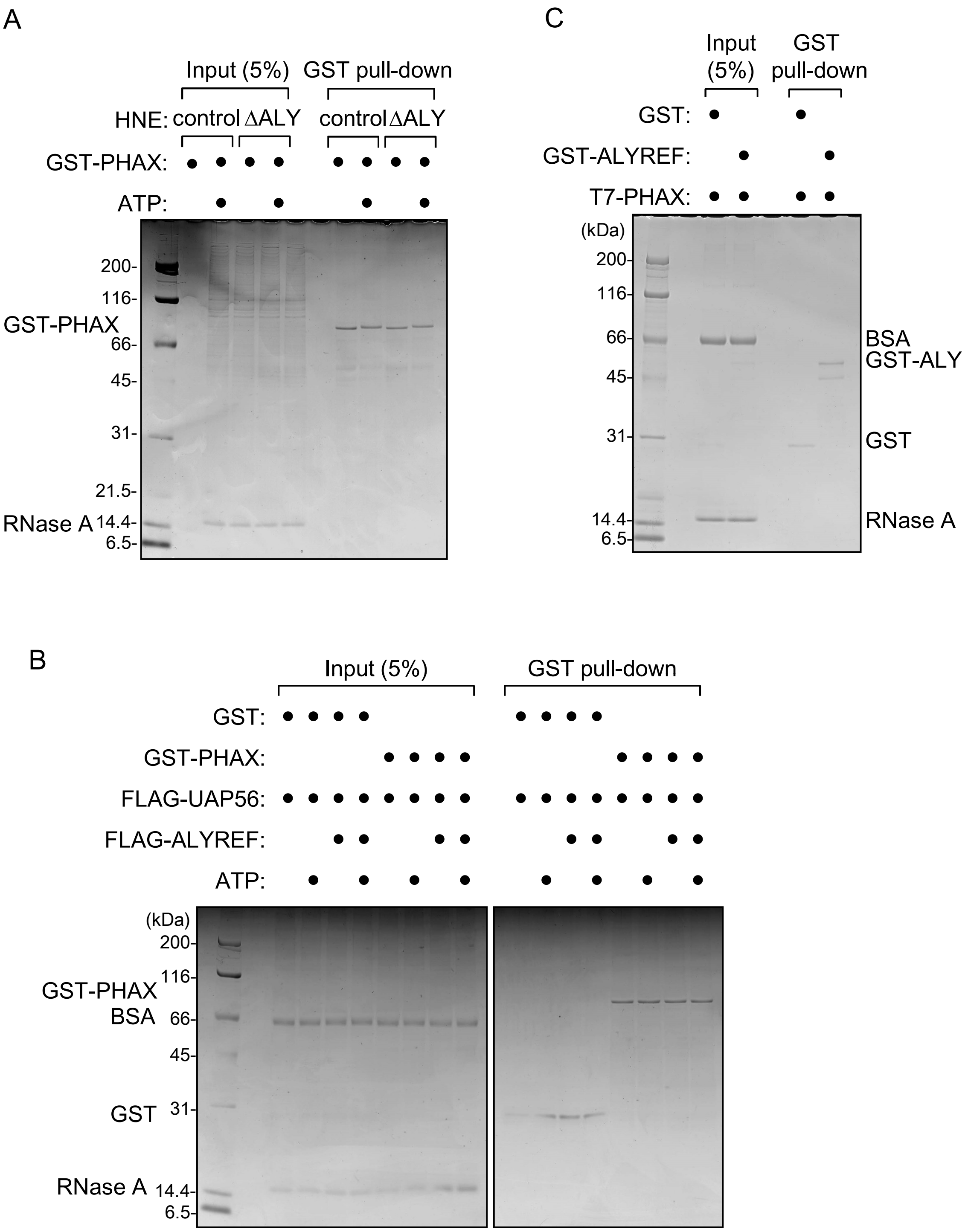

Supplementary Figure S7: Coomassie brilliant blue (CBB) staining of Figure 6.
(A—C) CBB staining of Figure 6A, 6B, and 6C.

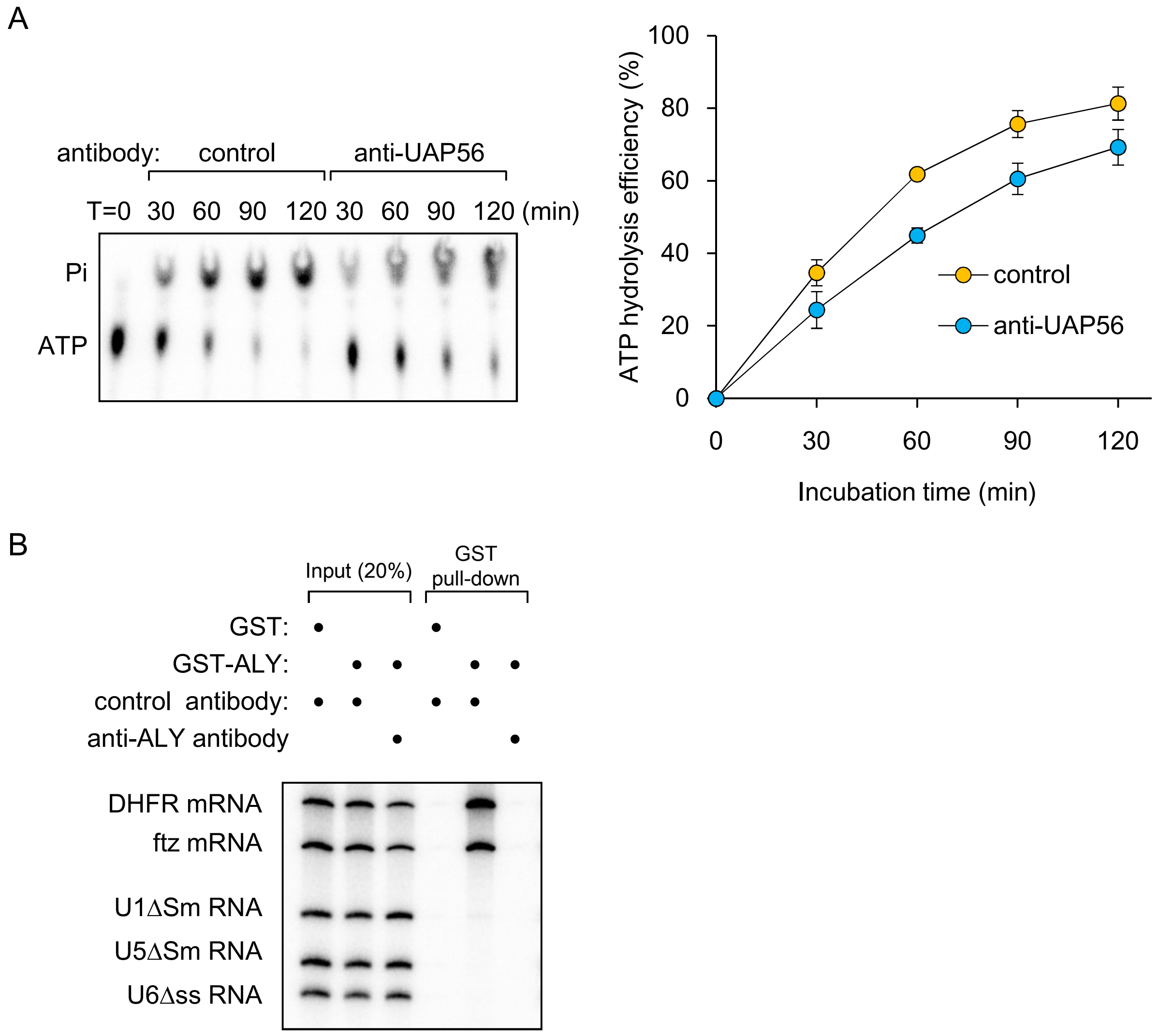

Supplementary Figure S8: Antibodies against UAP56 and ALY.
(A) Purified recombinant FLAG-UAP56 and [γ-^{32}P] ATP were incubated with an anti-UAP56 antibody or a control antibody at 37°C. Products were developed by thin layer chromatography. ATP and inorganic phosphate (Pi) were detected by autoradiography. Quantification of ATP hydrolysis efficiency from three independent results is shown.
(B) A mixture of *in vitro*-transcribed ^{32}P-labeled RNAs containing DHFR mRNA, ftz mRNA, U1ΔSm, U5ΔSm, and U6Δss snRNAs was incubated with purified GST-ALY and either a control antibody or an anti-ALY antibody. Then, a GST pull-down assay was performed. Pulled-down RNAs were analyzed by denaturing PAGE and autoradiography.

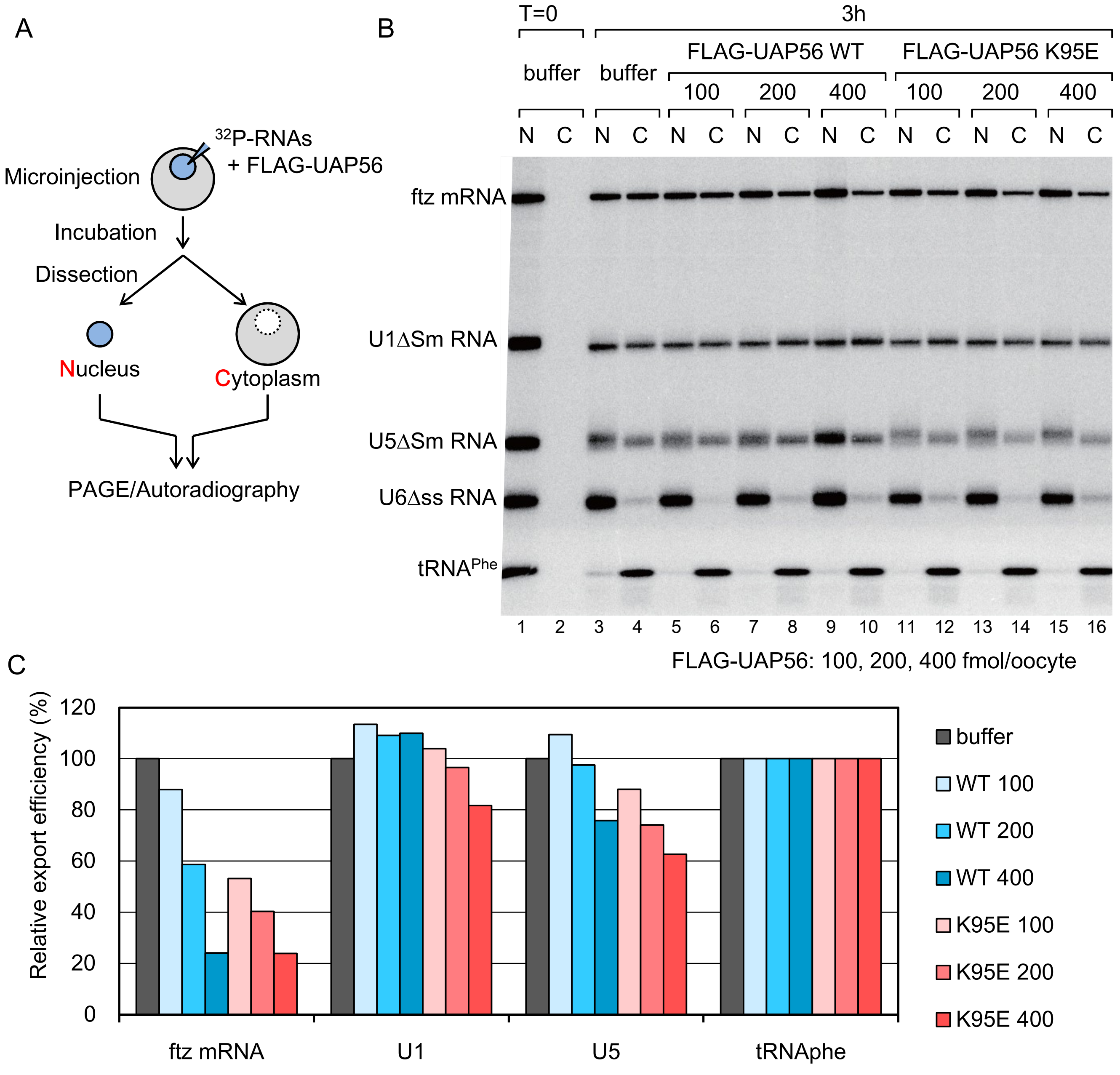

Supplementary Figure S9: Role of ATP-binding activity of UAP56 in mRNA and U small nuclear RNA (snRNA) export.

(A and B) ^{32}P-labeled RNAs were injected with or without purified recombinant FLAG-UAP56 WT or K95E mutant protein (100, 200, 400 fmol/oocyte) into the nucleus. After an incubation at 19°C for 3 hours, oocytes were dissected into nuclear and cytoplasmic fractions. RNA was extracted from each fraction and was analyzed by denaturing polyacrylamide gel electrophoresis (PAGE) and autoradiography.

(C) Quantification of the relative export efficiency of fushitarazu (ftz) mRNA, U1ΔSm RNA, U5ΔSm RNA, and $tRNA^{Phe}$. Export efficiency of the buffer control was set to 100% and each $tRNA^{Phe}$ export efficiency was set to 100%.

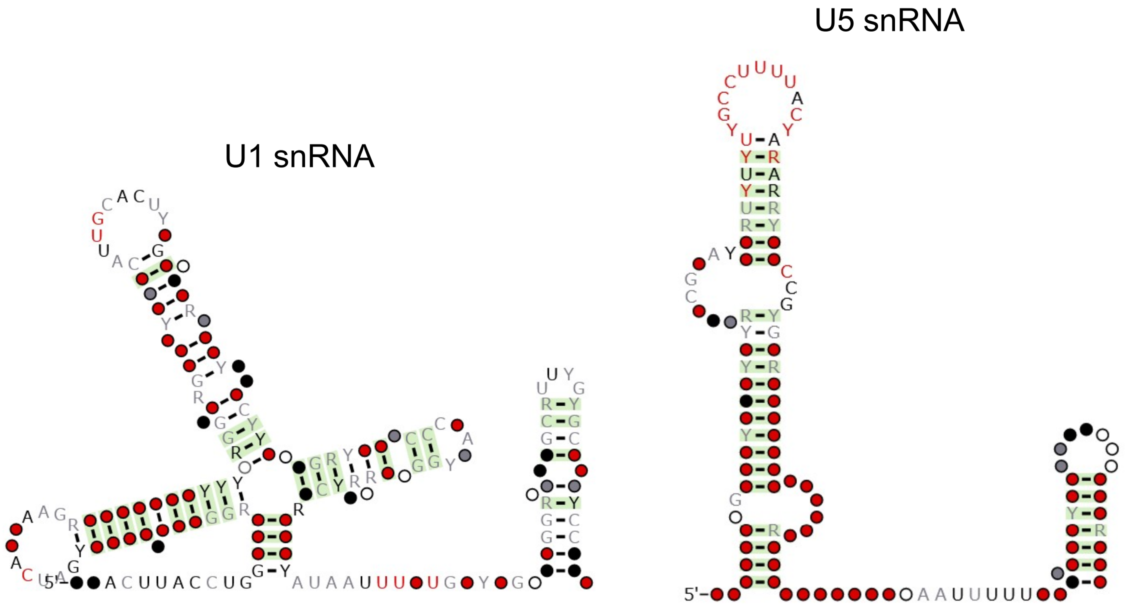

Supplementary Figure S10: Secondary structure of U1 and U5 small nuclear RNAs (snRNAs).

The images of secondary structure of U1 (RF00003) and U5 (RF00020) snRNAs were obtained from the Rfam database.
